# Different Pretreatment Methods to Strengthen the Microwave Vacuum Drying of *Honeysuckle*: Effects on the Moisture Migration and Physicochemical Quality

**DOI:** 10.3390/foods13223712

**Published:** 2024-11-20

**Authors:** Xiaoping Yang, Zhengying Ma, Fangxin Wan, Ao Chen, Wenkang Zhang, Yanrui Xu, Zepeng Zang, Xiaopeng Huang

**Affiliations:** College of Mechanical and Electronical Engineering, Gansu Agricultural University, Lanzhou 730070, China; yangxp@gsau.edu.cn (X.Y.); zangzp@st.gsau.edu.cn (Z.Z.)

**Keywords:** rotary microwave vacuum drying, *Honeysuckle*, pretreatment, drying characteristics, quality

## Abstract

In this study, we analyzed the effects of three pretreatment methods—microwave, steam, and blanching—on the quality of *Honeysuckle* to determine the optimal pretreatment method; we then investigated the influence of different drying temperatures, vacuum levels, and rotation speeds on the drying characteristics, color, and active ingredient content of the *Honeysuckle* that was pretreated by the optimal pretreatment method during rotary microwave vacuum drying. The results indicated that a microwave pretreatment for 75 s was the optimal pretreatment method, which enhanced the retention of active ingredients and effectively improved the browning of the material. During the process of rotary microwave vacuum drying, as the temperature increased, the vacuum level rose, and the rotation speed increased, the drying rate gradually increased. However, excessively high vacuum levels and rapid rotation speeds could actually decrease the drying rate. In addition, the total phenols, total flavonoids, antioxidant activity, and various active ingredients of *Honeysuckle* dried by rotary microwave vacuum were effectively preserved. Furthermore, its rehydration properties and color were significantly superior to those dried through sun drying. The TIOPSIS method analysis showed that the optimal process parameters were a temperature of 50 °C, a vacuum level of −0.070 MPa, and a rotation speed of 35 Hz, which exhibited the highest relative closeness (0.76). The comprehensive analysis indicated that microwave pretreatment followed by rotary microwave vacuum drying was a promising drying method with potential applications in the dehydration of agricultural products and medicinal plants.

## 1. Introduction

*Honeysuckle* (also known as *Flos lonicerae*) refers to the dried flower buds or newly opened flowers of Lonicera japonica Thunb, a plant belonging to the *Caprifoliaceae* family. It is widely distributed around the world [1]. *Honeysuckle* is rich in biologically active substances such as organic acids, flavonoids, iridoid glycosides, polysaccharides, and other compounds. It possesses a variety of functions including anti-inflammatory, antipyretic, antiviral, antioxidant, anticancer, hepatoprotective, hypoglycemic, neuroprotective, and immune-enhancing properties [2]. Because of its unique medicinal and health care value, it is loved by consumers and widely used in food, beauty and pharmaceutical industries. Fresh *Honeysuckle* tissue is delicate and has high moisture content. After harvesting, they are highly prone to browning, which leads to rapid deterioration in their physical and chemical properties, ultimately reducing their nutritional and commercial value [3]. Drying is one of the most effective methods for extending the shelf life of agricultural products and medicinal plants. It preserves the quality of the products by reducing their moisture content, thereby inhibiting the growth of microorganisms and chemical changes during storage [4].

Flower and leaf materials are highly sensitive to temperature and are prone to significant deterioration during the dehydration process. Common drying methods for *Honeysuckle* include sun drying and hot air drying. Sun drying is cost-effective but cannot guarantee quality and hygiene, which can affect the quality of the dried products. Although hot air drying is faster and easier to manage, its higher temperature can lead to a decrease in the color and content of active ingredients in the dried product. Various drying techniques, including freeze-drying, vacuum far-infrared drying, and gas-controlled heat pump drying, have been applied to the drying of *Honeysuckle* [5,6,7]. However, these methods suffer from drawbacks such as long drying cycles, low production efficiency, uneven drying, weak adaptability to the production site, and high energy consumption. Therefore, new technologies and applications that can improve drying rates, product quality, and cost savings have attracted significant attention.

Microwave vacuum drying represents a combined drying technology with significant potential for broad applications. Microwaves can penetrate into the interior of the material, causing polar molecules such as water inside the material to rotate synchronously and generate frictional heat, thereby rapidly heating the material from the inside out. Under vacuum conditions, the boiling point and oxygen content of water decrease, making the sample less prone to oxidation and thermal degradation. Therefore, microwave vacuum drying is characterized by short heating times, fast drying rates, and the ability to better preserve heat-sensitive components [8]. It has been widely used for drying various materials such as vegetables, fruits, medicinal herbs, meat, tea, and others [9,10,11,12,13]. Additionally, the non-thermal effects of microwaves also have bactericidal effects, which can significantly reduce the infestation rate of dried products during subsequent storage [14]. Currently, microwave vacuum drying is typically carried out as a continuous drying process. While it offers good drying performance, it has higher energy consumption and can suffer from uneven drying. This can lead to localized overheating, causing surface clumping and the scorching of the material. However, rotary microwave vacuum drying, being an intermittent drying process, can minimize heat damage and quality degradation. While maintaining good drying performance, it has the potential for energy savings, resulting in low energy consumption and high-quality dried products.

During the drying process of flower and leaf materials, as well as fruits and vegetables, enzymatic browning can easily occur. This can lead to undesirable changes in the color of the dried products and a reduction in the retention of phenolic and flavonoid antioxidants. However, employing appropriate pretreatment methods can inhibit enzyme activity and enhance the quality of the dried products. Araujo et al. conducted steam pretreatment on Galega Kale and found that it can improve the retention of vitamin C, total antioxidant capacity, as well as the appearance parameters and color of the dried product [15]. Liu et al. conducted different pretreatments on purple flesh sweet potato and found that microwave pretreatment can rapidly degrade peroxidase, leading to an increase in the retention of anthocyanins in the dried product [16]. On the other hand, blanching and steam pretreatment were found to better preserve the appearance and color of the dried product.

Therefore, to further improve the drying quality and application value of *Honeysuckle*, this study adopted three pretreatment methods: microwave, steam, and blanching, to investigate their effects on the quality of *Honeysuckle* dried using a rotary microwave vacuum drying method. The goal was to determine the optimal pretreatment method. Building on this, the study aimed to investigate the impact of rotary microwave vacuum drying on the drying characteristics of *Honeysuckle* and to explore the effects of different drying parameters on the color, rehydration capacity, total phenols, total flavonoids, polysaccharides, antioxidant capacity, and retention rates of seven active ingredients in *Honeysuckle* dried products. The study used the entropy weight-TOPSIS method for objective evaluation to identify the optimal drying process.

## 2. Materials and Methods

### 2.1. Test Material

The *Honeysuckle* used in the study was harvested in June 2023 from the *Honeysuckle* Plantation in Tongwei County, Dingxi City (Gansu, China), and the variety was “Beihua No. 1”. We were required to use untreated *Honeysuckle* without any processing. The *Honeysuckle* was purchased without any treatment and stored in a refrigerator at a temperature of 4 °C. According to the official AOAC method, the average moisture content of fresh *Honeysuckle*, determined by maintaining it at 105 °C for 24 h, was 77.47% [17].

### 2.2. Rotary Microwave Vacuum Drying Equipment

**Figure 1:**
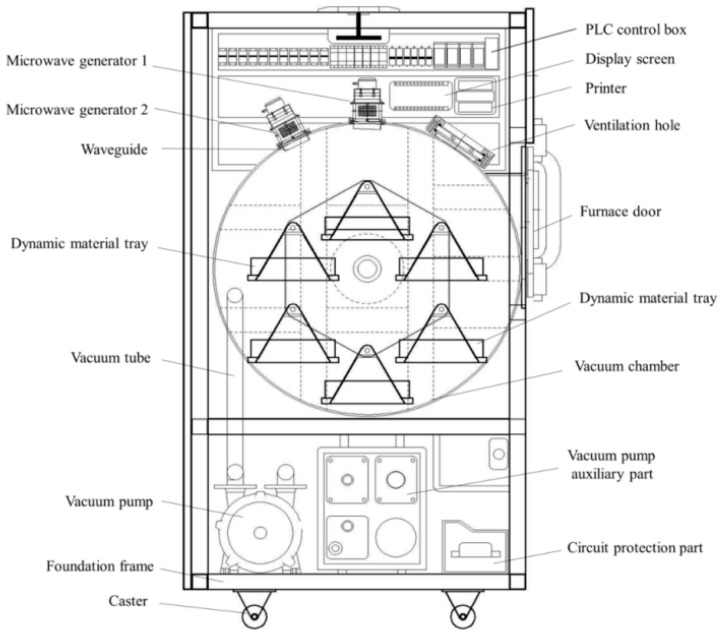
Schematic diagram of rotary microwave vacuum drying equipment.

The rotary microwave vacuum drying equipment used in this experiment (YZWS-CT-12 model, Tianshui Shenghua Microwave Technology Co., Ltd., Tianshui, China) is consistent with the equipment used in the experiment conducted by Zang et al. [18]. Its structure is depicted in Figure 1. The equipment features a microwave emitter located at the top, and there are six rotating tray racks inside the drying chamber. When the trays rotate to the top position, the microwave acts on the material, and this action is intermittent. By adjusting the speed of the rotating wheel, the intermittent microwave action time can be controlled.

### 2.3. Experimental Method

#### 2.3.1. Different Pretreatment Experiments

Uniformly sized samples with consistent moisture content and without apparent damage were selected as the experimental materials. Each pretreatment experiment was conducted in triplicate, with a sample weight of 60 ± 0.05 g per trial. The pretreatment method is as follows: (1) Microwave Pretreatment: microwave pretreatment was conducted using a microwave oven (Midea PC 20 W 3, Midea Kitchen Appliances Manufacturing Co., Ltd., Zhuhai, China) with a microwave power of 616 W; (2) Steam Pretreatment: the samples were placed in a metal basket and subjected to steam pretreatment in a steam jacketed vessel with a cover, where the steam temperature was maintained at 95 °C; (3) Hot Water Blanching Pretreatment: hot water blanching pretreatment was conducted in an electrically heated constant temperature water bath (HHS 21-6, Shanghai Boxun Medical Biological Instruments Co., Ltd., Shanghai, China), with the hot water temperature set at 95 °C.

The effects of different pretreatments on *Honeysuckle* were studied at four levels: 30 s, 45 s, 60 s, and 70 s. Preliminary experimental results are presented in Table 1.

When the material does not undergo browning, it indicates that its internal oxidase activity has been significantly inhibited. Further extending the pretreatment time may lead to the loss of medicinal and nutritional components within the material. Therefore, the final selected pretreatment methods are as in Table 1. After high-temperature pretreatment, the samples were immediately flattened and cooled down to avoid yellowing caused by the accumulation of heat. For samples treated with hot water blanching, a paper towel was used to wipe off surface moisture.

The dried samples were spread out on the tray and placed into a rotary microwave vacuum dryer. The drying conditions were set at 50 °C, −0.065 MPa, and 35 Hz to dry the samples to a safe moisture content. The study aimed to investigate the effects of different pretreatment methods on the quality of *Honeysuckle* dried by microwave vacuum drying and to determine the optimal pretreatment method.

The control test here was chosen to be microwave vacuum drying of unpretreated samples at 50 °C, −0.065 MPa, and 35 Hz.

The control test was selected as sun drying (Sun). For Sun: Spread *Honeysuckle* in a single, even layer on a drying tray and place outdoors exposed to sunlight, recording the weight of the *Honeysuckle* every 3 days until the moisture content drops below the safe moisture content.

#### 2.3.2. Rotary Microwave Vacuum Drying Experiment

This experiment was performed in order to explore the influence law on the drying characteristics and quality of *Honeysuckle* under different rotary microwave vacuum parameters. After selecting the best pretreatment method according to the pretreatment test, the rotary microwave vacuum drying equipment was used to carry out the subsequent drying test; the selection of test factors is shown in Table 2. Before drying, the equipment was preheated for 30 min with specific parameters set in advance, the pretreated and cooled *Honeysuckle* was spread on the material tray and put on the material tray rack for drying, its weight was measured once every 90 s until the moisture content of the material fell below the safe moisture content, and then the drying was stopped.

### 2.4. Calculation of Experimental Indicators

#### 2.4.1. Calculation of Dry Basis Moisture Content

(1) $$X=\frac{M_{t}-M_{d}}{M_{t}}$$

where *X* represents the moisture content on a dry basis, %; *M_t_* represents the weight of the *Honeysuckle* at time, g; and *M_d_* represents the weight of the dried *Honeysuckle* product, g.

#### 2.4.2. Calculation of Drying Rate

(2) $$DR=\frac{M_{t1}-M_{t_{2}}}{t_{2}-t_{1}}$$

where *DR* represents the drying rate, g/s;$\;M_{t1}\;and\;M_{t2}$ represent the weight of the *Honeysuckle* at times *t*_2_ and *t*_1_, g; and *t*_2_ − *t*_1_ represents the time interval between two weighings during the drying process.

#### 2.4.3. Calculation of Moisture Ratio

(3) $$MR=\frac{X_{t}-X_{e}}{X_{c}-X_{e}}$$

where *MR* represents the moisture ratio; *X*_t_ represents the moisture content on a dry basis at time, %; *X*_e_ represents the equilibrium moisture content of the *Honeysuckle*, %; and *X*_c_ represents the initial moisture content of the *Honeysuckle*, %.

### 2.5. Determination of Rehydration Ratio

The dried *Honeysuckle* was soaked in distilled water at 40 °C for rehydration. After weighing 2 g of sample and adding it to 150 mL of distilled water, the samples were removed at 60 min, 180 min, and 300 min intervals. Any surface water was then drained using filter paper, and the mass of each sample was recorded [19]. Each experimental group was repeated three times to ensure accuracy and reliability. The formula for calculating the rehydration ratio was as follows:

(4) $$R_{f}=\frac{G_{f}}{G_{g}}$$

where R*_f_* represents the rehydration ratio; *G_f_* represents the mass of the surface moisture removed after rehydration, g; and *G_g_* represents the mass of the dried *Honeysuckle* before rehydration, g.

### 2.6. Determination of Color

The color evaluation of *Honeysuckle* was conducted using a precision colorimeter (CS-210, Zhengzhou Nanbei Instrument Equipment Co., Ltd., Zhengzhou, China). The measurement results were represented by L*, a*, and b* values, and the total color difference (∆E) was calculated according to the following Formula (5):

(5) $$∆E=\sqrt{{\left(L^{*}-L_{0}\right)}^{2}+{\left(a^{*}-a_{0}\right)}^{2}+{\left(b^{*}-b_{0}\right)}^{2}}$$

where ∆E represents the total color difference in the sample; L*, a*, and b* represent the lightness, red/green value, and yellow/blue value of the fresh *Honeysuckle* sample, respectively; and *L*, *a*_0_, and *b*_0_ represent the lightness, red/green value, and yellow/blue value of the dried *Honeysuckle* product, respectively.

### 2.7. Determination of Polysaccharide Content

Following the method described by Dubious et al. [20], the determination of polysaccharide content involved calibrating with sucrose as the standard. Each experiment was repeated three times. The polysaccharide content was calculated using Formula (6), and the sucrose standard curve equation was represented by Formula (7).

(6) $$\mathrm{Polysaccharide}\;\mathrm{content}=\frac{V_{2}C_{1}}{V_{1}M}$$

(7) $$\mathrm{Standard}\;\mathrm{curve}\;\mathrm{equation}\;Y=0.091X-0.006\left(R^{2}=0.9866\right)$$

where *C*_1_ represents the calculated concentration of polysaccharide based on the standard curve, mg/mL; *V*_1_ is the volume of the sample extraction solution during titration, mL; *V*_2_ is the total volume of the sample extraction solution, mL; and *M* is the mass of the dried *Honeysuckle* sample, g.

### 2.8. Determination of Total Phenolic Content

Following the method described by Beato et al. [21], the determination of total phenolic content involved calibrating with gallic acid as the standard. Each experiment was repeated three times. The total phenolic content was calculated using Formula (8), and the gallic acid standard curve equation was represented by Formula (9).

(8) $$\mathrm{Total}\;\mathrm{phenolic}\;\mathrm{content}=\frac{V_{2}C_{2}}{V_{1}M}$$

standard curve equation for gallic acid y = 2.479x − 0.047*R*^2^ = 0.9993 (9)

where *C*_2_ represents the calculated concentration of total phenols based on the standard curve, mg/mL; *V*_1_ is the volume of the sample extraction solution during titration, mL; *V*_2_ is the total volume of the sample extraction solution, mL; and *M* is the mass of the dried *Honeysuckle* sample, g.

### 2.9. Determination of Total Flavonoid Conten

Referencing the measurement method of Lay et al. [22], catechin was used as the standard substance for calibration, with each experimental group repeated three times. According to Formula (10), the total flavonoid content was calculated, with the catechin standard curve equation represented by Formula (11).

(10) $$\mathrm{Total}\;\mathrm{flavonoid}\;\mathrm{content}\;=\frac{V_{2}C_{3}}{V_{1}M}$$

standard curve equation for catechin y = 0.216x − 0.006*R*^2^ = 0.9973 (11)

where *C*_3_ is the calculated concentration of total flavonoids based on the standard curve, mg/mL; *V*_1_ is the volume of the sample extraction solution during titration, mL; *V*_2_ is the total volume of the sample extraction solution, mL; and *M* is the mass of the dried *Honeysuckle* sample, g.

### 2.10. Determination of Antioxidant Capacity

Referencing the measurement method of Nencini et al. [23], each experimental group was repeated three times. The antioxidant capacity was calculated using Formula (12):

(12) $$\mathrm{Inhibition}\;\mathrm{rate}=\frac{\left(A_{0}-A\right)}{A_{0}}\times 100\%$$

where *A* is the absorbance of the sample solution; and *A*_0_ is the absorbance of the blank control.

### 2.11. Determination of Active Ingredients Contents

#### 2.11.1. Chromatographic Conditions

The active ingredients contents analysis of the dried samples was carried out using an HPLC (Agilent 1100, Agilent Technology Co., Ltd., Santa Clara, CA, USA) column, with the following parameters: Merk RP-C18 (250 mm × 4.6 mm, 5 μm); column temperature 38 °C; injection volume 10 μL; flow rate 1.0 mL/min; gradient elution: mobile phase acetonitrile (A): −0.2% formic acid water (B): 0–7 min, 10–15% A; 7–10 min, 15–16% A; 10–16 min, 16–20% A; 16–20 min, 20–22% A; 20–23 min, 22–24% A; 23–25 min, 24–30% A; 25–27 min, 30–38% A; 27–30 min, 38–8% A; 30–32 min, 8–8% A; detection wavelength 252 nm [24].

#### 2.11.2. Preparation of Reference Solution

For the preparation of the reference solution, we accurately weighed the appropriate amounts of chlorogenic acid, luteoloside, rutin, loganin, isochlorogenic acid A, isochlorogenic acid C, and quercetin reference standards, added an appropriate amount of methanol, and prepared individual reference standard solutions with a mass concentration of 1 mg/mL for each compound. An appropriate amount of each individual reference standard solution was taken, followed by the addition of methanol, to prepare mixed standard solutions with concentrations of 0.125 mg/mL, 0.0625 mg/mL, 0.03125 mg/mL, 0.015625 mg/mL, 0.0078125 mg/mL, and 0.00390825 mg/mL, respectively, which were then thoroughly mixed.

#### 2.11.3. Preparation of Sample Solution

The dried *Honeysuckle* was ground and sieved through a 0.177 mm mesh size. Then, 0.5 g of the sample was accurately weighed and placed into a conical flask. Next, 25 mL of 50% methanol was added, and the mixture was subjected to 30 min of ultrasonic extraction (power: 320 W, frequency: 40 kHz). After cooling, the weight loss was compensated for by adding 50% methanol. The mixture was shaken well, filtered, and the filtrate was passed through a 0.22 μm microporous membrane.

### 2.12. Optimization of Drying Process

The entropy weight method is a multi-attribute objective decision-making approach that determines weights based on the variability of each indicator. In this method, higher variability results in greater weight, indicating higher importance in the evaluation system. The TOPSIS (Technique for Order of Preference by Similarity to Ideal Solution) method is a ranking technique used to approximate the ideal solution. When the drying conditions are closest to the ideal solution, it represents the optimal processing method [25]. By combining the entropy weight method with the TOPSIS method, the optimal process parameters for the microwave vacuum drying of *Honeysuckle* can be determined. The calculation steps are as follows:

#### 2.12.1. Calculate the Entropy Value e_j_ for Indicator I_j_

(13) $$e_{j}=-\frac{1}{\ln n}\overset{m}{\underset{i=1}{\sum }}p_{\mathrm{ij}}\ln p_{\mathrm{ij}}$$

where m is the number of drying schemes for cherries; n is the number of indicators for each scheme; and p_ij_ is the characteristic weight of the j indicator for the i-th scheme.

#### 2.12.2. Calculate the Weight w_j_ of Indicator I_j_

(14) $$w_{j}=\frac{d_{j}}{\sum_{j=1}^{n}d_{j}}$$

where d_j_ is the utility value of the j-th indicator.

#### 2.12.3. Calculate the Positive and Negative Ideal Solutions for Indicator I_j_


(15) $$z^{+}=\left(z_{1}^{+},z_{2}^{+},\;\ldots ,\;z_{n}^{+}\right)$$

(16) $$z^{-}=\left(z_{1}^{-},\;z_{2}^{-},\;\ldots ,\;z_{n}^{-}\right)$$

where $z_{j}^{+}=\max\left\{z_{\mathrm{ij}}\right\};\;z_{j}^{-}=\min\left\{z_{\mathrm{ij}}\right\}$.

#### 2.12.4. Calculate the Euclidean Distance Between the Positive and Negative Ideal Solutions for Each Evaluation Indicator I_j_

(17) $$\left\{\begin{array}{c} D_{i}^{+}=\sqrt{\overset{n}{\underset{j=1}{\sum }}{\left(z_{j}^{+}-z_{\mathrm{ij}}\right)}^{2}}\\ D_{i}^{-}=\sqrt{\overset{n}{\underset{j=1}{\sum }}{\left(z_{\mathrm{ij}}-z_{j}^{-}\right)}^{2}} \end{array}\right.$$

where $D_{i}^{+}$ is the positive ideal solution for the i-th scheme and where $D_{i}^{-}$ is the negative ideal solution for the i-th scheme.

#### 2.12.5. The Closeness Coefficient C_i_ Between the Evaluated Scheme and the Ideal Value

(18) $$C_{i}=\frac{D_{i}^{-}}{D_{i}^{-}+D_{i}^{+}}$$

where the range of $C_{i}$ is [0,1], and the higher the value of $C_{i}$, the higher the comprehensive score, scheme $S_{i}$ is selected as the optimal choice based on its comprehensive comparison ratio.

### 2.13. Statistical Analysis

Each experiment was repeated three times, and the data were processed using Excel 2019 software. The results were presented as the mean ± standard deviation. Analysis of variance (ANOVA) was performed using SPSS 27.0 software (*p* < 0.05). Graphs were plotted using Origin 2022.

## 3. Results and Discussion

### 3.1. Effect of Different Pretreatment Processes on the Quality of Microwave Vacuum Dried Honeysuckle

**Figure 2:**
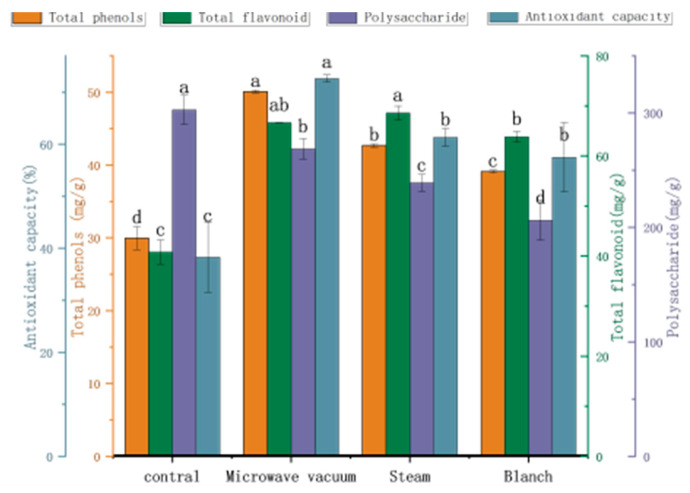
Effect of different pretreatment methods on the total phenolic content, total flavonoid content, polysaccharide content, and antioxidant capacity. Note: A different lowercase letter after each column indicates a significant difference (*p* < 0.05).

As shown in Figure 2 and Table 3, various pretreatments notably improved the retention of total phenolics, total flavonoids, antioxidant capacity, and several active constituents (such as chlorogenic acid, loganin, rutin, luteoloside, isochlorogenic acid A, isochlorogenic acid C) in *Honeysuckle* (*p* < 0.05). This suggests that microwave, steam, and blanching pretreatments effectively suppressed peroxidase activity, reducing enzyme-catalyzed reactions and thus preserving the total phenolic and flavonoid compounds that served as substrates for these reactions. However, the polysaccharide content significantly decreased after pretreatment (*p* < 0.05). The Maillard reaction is more likely to occur during high-temperature pretreatment due to increased contact between the material and oxygen, in comparison to rotary microwave vacuum drying. Han et al. [26] observed that the polysaccharide content of dried Bletilla striata scented tea decreased after pretreatment. A significant analysis of the three pretreatment methods revealed that microwave pretreatment resulted in significantly higher retention rates of total phenolics, polysaccharides, antioxidant capacity, chlorogenic acid, and isochlorogenic acid A compared to the other pretreatment methods (*p* < 0.05), with values of 50.03 mg/g, 268.67 mg/g, 72.65%, 44.73 mg/g, and 11.24 mg/g, respectively.

**Figure 3:**
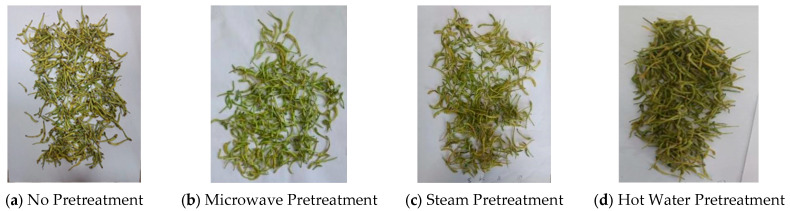
Images of dried *Honeysuckle* after different pretreatments.

The macroscopic structure of *Honeysuckle* after different pretreatments was shown in Figure 3. The large and middle ends of the flowers without pretreatment that were dried by rotary microwave vacuum darkened, which was effectively avoided with pretreatment. Table 4 shows that after pretreatment, the brightness of the material increased and the greenness, yellowness, and overall color difference decreased. Material treated with microwave pretreatment combined with rotary microwave vacuum drying exhibited significantly increased brightness compared to other treatment methods, with a less pronounced decrease in greenness. Based on the comprehensive analysis above, it was concluded that microwave pretreatment better preserved the content of active ingredients compared to steam and blanching pretreatment methods. Additionally, the appearance and color of the material treated with microwave pretreatment were greener. Furthermore, the microwave pretreatment stage could evaporate some moisture from the material, thus saving subsequent drying costs. Therefore, microwave pretreatment was chosen for the pretreatment of *Honeysuckle* in this experiment.

### 3.2. Drying Characteristics

#### 3.2.1. Effect of Drying Temperature on the Drying Characteristics

**Figure 4:**
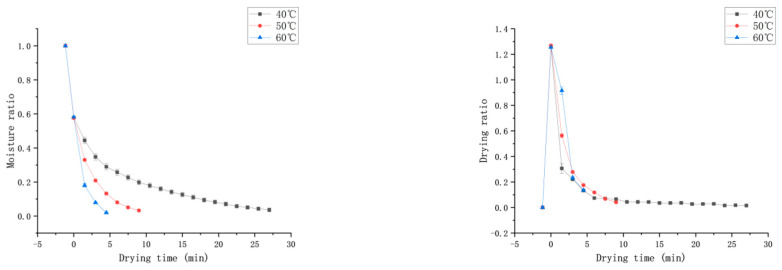
Effect of different drying temperatures on the moisture ratio (MR) and drying rate (DR).

Figure 4 depicts the drying characteristic curves of *Honeysuckle* at different temperatures under a vacuum degree of −0.060 MPa and a rotation speed of 35 Hz. The curve indicates that as the temperature increased, the moisture content of the *Honeysuckle* gradually decreased, and the drying rate accelerated. At 60 °C, the drying time was the shortest (4.5 min), which was 83.3% shorter than that at 40 °C (27 min). This indicates that increasing the drying temperature had a positive effect on reducing the dehydration time. This may be due to the fact that with the increase in microwave temperature, the steam inside the material will form a huge driving force so that the moisture is transferred to the surface of the material in the form of water molecules, and a large pressure difference is formed, which promotes the diffusion of moisture from the inside of the material to the surface and accelerates the rate of moisture transfer. The direction of heat transfer of the material in the microwave field and the direction of diffusion of the temperature gradient is the same, which accelerates the rate of moisture migration and shortens the drying time [27]. Moreover, as temperature increased, the mobility and turbulence of the water molecules were enhanced, which led to an increase in microporosity and a reduction of tissue structure, a reduction in mass transfer resistance and the acceleration of the migration rate of the water [28]. As the drying process progressed, free water was initially completely removed, and the content of bound water continued to decrease. During this process, the binding force and mass transfer resistance of internal water in the material gradually increased, making dehydration more difficult and reducing the drying rate.

#### 3.2.2. Effect of Vacuum Pressure on the Drying Characteristics

**Figure 5:**
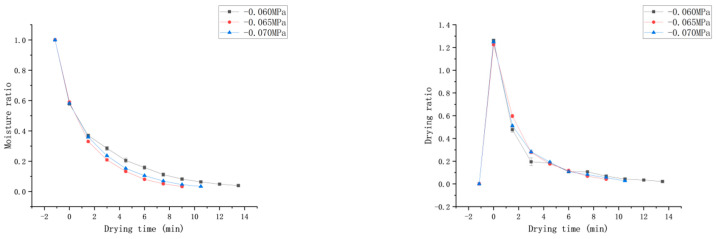
Effect of different vacuum levels on the drying characteristics.

Figure 5 illustrates the drying characteristic curves of *Honeysuckle* at different vacuum pressures at 50 °C and 35 Hz. Observing the curves, it was evident that with the increase in vacuum pressure, the moisture ratio rate of the sample initially increased and then decreased. When the vacuum pressure was −0.065 MPa, it only took 9 min for the sample to reach the safe moisture level, which was shorter by 33.3% than that at −0.060 MPa. And the drying time was the shortest under this condition, likely due to the increased vacuum, which enhanced the pressure gradient between the interior and exterior of the sample. This gradient elevated the internal energy and activity of the water molecules, thereby accelerating moisture migration and reducing drying time. Additionally, with the increasing of the vacuum pressure, the boiling point of water was low and the evaporation of moisture was faster [29]. However, when the vacuum pressure was −0.070 MPa, the drying time was longer by 16.7% compared to −0.065 MPa, which could be due to the excessively high vacuum pressure leading to a longer time needed to heat the machine, and a reduction in energy absorbed by the sample; therefore, the drying rate was decreased. Similar trends have been observed by Liu et al. [30] in the drying of garlic and by Zang et al. [18] in the drying of *Angelica sinensis*.

#### 3.2.3. Effect of Rotation Speed on the Drying Characteristics

**Figure 6:**
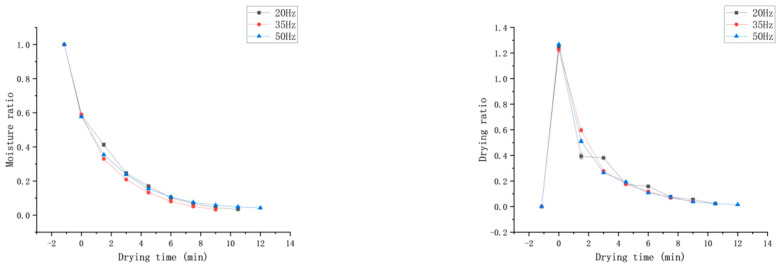
Effect of different rotation speeds on the drying characteristics.

Figure 6 illustrates the drying characteristic curves of *Honeysuckle* at different rotation speeds under a drying temperature of 50 °C and a vacuum level of −0.065 MPa. Observing the curve, it is evident that with the increase in the rotational speed of the drying equipment, the moisture rate of the sample change first increased and then decreased. This may be due to the fact that at rotational speeds of 20 Hz, 35 Hz, and 50 Hz, the material was exposed to microwave radiation for 20 s, 12 s, and 8 s, respectively, with intermittent periods of 100 s, 60 s, and 40 s. The increase in the intermittent period could facilitate the diffusion of moisture to the material surface, saving energy. However, if the intermittent period was too long, it could cause a decrease in material temperature, slowing down evaporation and consequently reducing the drying rate [31]. At 35 Hz, the energy supplied by microwave was most closely matched to the energy required for drying, and in the drying rate was highest. Increasing the intermittent time had a greater impact on the initial stage of drying and a smaller impact on the later stage. This could be due to the fact that during the initial stage of drying, the sample gains high sensible heat at high temperatures, causing the moisture to rapidly redistribute and migrate to the surface [32]. In the later stages of drying, when the moisture content of *Honeysuckle* fell below 0.33 g water/g solid, its microwave absorption efficiency decreased with decreasing moisture content. This finding is consistent with the results of Su et al. [33].

### 3.3. Drying Qquality

#### 3.3.1. Color

Based on Table 5, the brightness (L*), red/green value (a*), and yellow/blue value (b*) of fresh *Honeysuckle* was 75.89, −11.97, and 31.71, respectively. After rotary microwave vacuum drying, their retention rates of L*, a*, and b* were 103.82% to 105.02%, 54.47% to 60.06%, and 81.49% to 84.14%, respectively. This indicates that the microwave pretreatment followed by rotary microwave vacuum drying could effectively retain the surface brightness of the material, but it might lead to a reduction in the green and yellow colors of *Honeysuckle* while increasing the red and blue colors. When compared to microwave vacuum drying, the overall color difference was larger, and brightness was improved significantly (*p* < 0.05) for *Honeysuckle* dried by sun. This could be because the diffusion of moisture was slow during low-temperature drying and so there was little damage to the cell structure and the regional distribution of enzymes and substrates was not broken. Consequently, only slight browning or minimal browning phenomena occurred for *Honeysuckle* dried by sun. The color of the *Honeysuckle* did not show significant changes under different drying conditions, which may be attributed to the significant inhibition of enzyme activity by microwave pretreatment, reducing the enzymatic browning process. Additionally, the high efficiency and short drying time for the rotary microwave vacuum drying method may result in the Maillard reaction only occurring in the initial and colorless intermediate stages, with the formation of colored substances not yet initiated or incomplete [34].

#### 3.3.2. Rehydration Ratio

The rehydration ratio of dried *Honeysuckle* under different drying conditions, as shown in Table 5, indicates that a higher rehydration ratio corresponds to less structural damage during the drying process. The rehydration capacity of *Honeysuckle* after microwave pretreatment followed by rotary microwave vacuum drying was significantly higher than that of the sun-dried *Honeysuckle*. This could be due to the severe wrinkling of the structure of sun-dried *Honeysuckle*, which increases the resistance to moisture transfer, leading to reduced rehydration capacity. Additionally, as the temperature increased, the rehydration ratio gradually increased. This could be due to the intensified movement of water molecules at higher temperatures, leading to an increase in micropores and a reduction in structural shrinkage, thus enhancing the rehydration capacity. This finding is consistent with the conclusion drawn by Guo et al. [35] in their study on hot air drying garlic. As the rotation speed and vacuum degree increased, the rehydration ratio showed an initial increase followed by a decrease. This could be attributed to the increase in vacuum degree leading to higher internal stress during the drying process, resulting in increased porosity within the material [36]. However, excessively high vacuum levels may disrupt the original internal structure of the material, leading to the observed trend in the rehydration ratio.

#### 3.3.3. Total Phenolic Content

**Figure 7:**
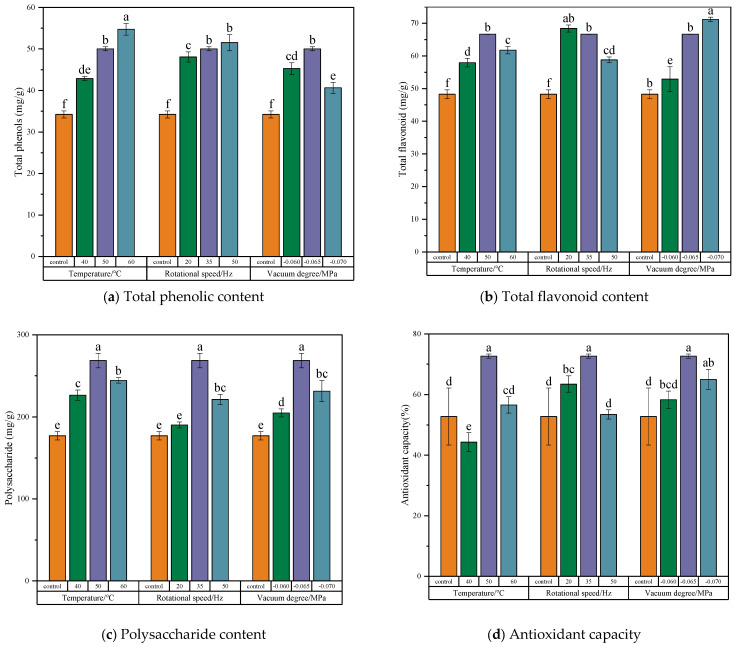
Effect of different drying conditions on the retention rate of total phenolic, total flavonoids, polysaccharides, and antioxidant capacity. Note: A different lowercase letter after each column indicates a significant difference (*p* < 0.05).

The influence of different drying conditions on the total phenolic content of *Honeysuckle* is illustrated in Figure 7a. At 60 °C, 35 Hz, and −0.065 MPa, the total phenolic content was highest (54.73 mg/g), significantly higher than that under other conditions (*p* < 0.05). Additionally, the total phenolic content under different drying conditions surpassed that by sun drying, indicating that microwave pretreatment followed by rotary microwave vacuum drying could effectively preserve phenolic compounds during the dehydration process of *Honeysuckle*. With the increase in drying temperature, the total phenolic content gradually increased. This may be due to the decreasing activity of polyphenol oxidase and peroxidase enzymes with increasing temperature. At 40 °C, the temperature and heightened enzyme activity could facilitate the oxidation and decomposition of phenolic substances, thereby decreasing their content. At 60 °C, enzyme activity was significantly inhibited and the total phenolic content was increased. This was similar to the conclusion drawn by Siddiq et al. [37] in their study on the activity of peroxidase in blueberries. As the rotation speed increased, the total phenolic content also increased, but the trend was relatively gradual, reaching 48.07 mg/g at 20 Hz, 50.03 mg/g at 35 Hz, and 51.51 mg/g at 50 Hz, respectively. As the vacuum degree increased, the total phenolic content gradually increased, reaching its highest level at −0.065 MPa (50.03 mg/g), after which it began to decrease. This could be because at higher vacuum levels, the enzymatic reactions of phenolic acids and enzymes with oxygen as the oxidant are reduced, resulting in a better retention of total phenolic content [7]. However, at excessively high vacuum levels, the cell walls of *Honeysuckle* may rupture, leading to disruption in the distribution of enzymes and phenolic substances within the cells, resulting in an increased loss of phenolic substances [38].

#### 3.3.4. Total Flavonoid Content

The impact of various drying conditions on the total flavonoid content of *Honeysuckle* was illustrated in Figure 7b. The total flavonoid content increased by 6.9% to 32% under different drying conditions when compared to sun drying. The highest total flavonoid content (71.14 mg/g) was observed at 50 °C, 35 Hz, and −0.070 MPa. The lowest total flavonoid content (52.87 mg/g) was observed at 50 °C, 35 Hz, and −0.060 MPa. This suggests that the vacuum degree positively influenced the retention of flavonoids in *Honeysuckle*. Similar results were observed by Zang et al. [18] during the drying of Angelica sinensis, which may be attributed to the increase in the vacuum degree preventing the non-enzymatic oxidation of flavonoids induced by heat treatment [39]. Observing the trend in total flavonoid content at different rotation speeds, it became evident that lower rotation speeds are more conducive to the retention of total flavonoids. The highest total flavonoid content (68.38 mg/g) was observed at 20 Hz. With the increase in temperature, there was an initial rise followed by a decline in total flavonoid content. This trend might be due to the promotion of the thermal degradation of cellulose and lignin in *Honeysuckle* by the rotary microwave vacuum drying (at 50 °C), which releases flavonoids from the insoluble parts of the plant, enhancing the extraction efficiency of total flavonoids and leading to the observed increase in content. However, a further increase in temperature promotes the thermal degradation of flavonoids, leading to a decrease in their content [40]. This observation is similar to the trend observed by Xu et al. [41] during the drying of goji berries.

#### 3.3.5. Polysaccharide Content

The influence of different drying conditions on the polysaccharide content of *Honeysuckle* was illustrated in Figure 7c. The polysaccharide content under different drying conditions was superior to that of sun-drying. As the drying temperature, rotation speed, and vacuum degree increased, the polysaccharide content in the dried *Honeysuckle* products exhibited a trend in initially increasing and then decreasing (*p* < 0.05). At 50 °C, 35 Hz, and −0.065 MPa, the polysaccharide content reached its peak at 268.67 mg/g. The pattern of increasing and then decreasing polysaccharide content with rising temperature may be attributed to the higher drying rate and a shorter drying time at elevated temperatures. This circumstance restricts the adequate reaction time for Maillard reactions with carbonyl compounds and amino compounds, which serve as substrates for these reactions. As a result, the polysaccharide components were better preserved under these conditions. However, at 60 °C, although the drying time was shortened, the higher temperature accelerated the rate of Maillard reactions [42]. which accelerated the decrease in polysaccharide content. This discovery parallels the findings of Jiang et al. [43] in their research on drying Cistanche slices. With increasing rotation speed, the polysaccharide content gradually increased. This could be because at 20 Hz, the *Honeysuckle* was exposed in hot air for a longer duration during the process, accelerating enzymatic and non-enzymatic browning reactions. This results in the lowest retention of polysaccharides (190.36 mg/g), only slightly higher than sun drying (177.16 mg/g). However, at 50 Hz, the moisture content reached a moderate state rapidly in the early stages. Subsequently, the drying rate decreased, extending the drying time. It was generally believed that the Maillard reaction was most likely to occur at a moderate moisture content state [44]. The extended drying time also allowed for sufficient reaction time, resulting in a decrease in the polysaccharide content.

#### 3.3.6. Antioxidant Capacity

The antioxidant capacity of *Honeysuckle* was assessed by the scavenging rate of the DPPH radical. The influence of different drying conditions on the antioxidant capacity of *Honeysuckle* is shown in Figure 7d. The figure indicates that with increasing temperature, the antioxidant capacity of *Honeysuckle* initially rose and then declined. At 50 °C, 35 Hz, and −0.065 MPa, the DPPH value reached peak at 72.65%. This trend might be due to the higher temperature shortened drying time mitigating the oxidative degradation of phenols and flavonoids components. However, at excessively high temperatures, oxidation and degradation for glycosides and quinones can occur more rapidly, leading to a decrease in the antioxidant capacity of *Honeysuckle*. It is indicated that increasing the drying temperature properly was beneficial to prevent the thermal damage and oxidative damage of bioactive compounds, so as to improve the antioxidant capacity of *Honeysuckle*. This was similar to the results of Zhang et al. [45] on dried wolfberry. With increasing rotation speed and vacuum level, the antioxidant capacity of *Honeysuckle* exhibited an initial enhancement followed by a decline. This trend did not consistently correspond to the variations in the content of total phenols, total flavonoids, polysaccharides, and other antioxidant components. This discrepancy may be due to the overall antioxidant activity being the combined result of the activity of each antioxidant component in the dried product. During the drying process, different antioxidant components can interact with each other, either synergistically or antagonistically [46].

#### 3.3.7. Active Ingredients Content

The impact of different drying conditions on the active components of *Honeysuckle* is shown in Table 6. Observing the table, under the conditions of 50 °C, 35 Hz, and −0.065 MPa, the dried *Honeysuckle* contained the highest levels of chlorogenic acid, rutin, and luteoloside at 44.73 mg/g, 2.84 mg/g, and 56.34 mg/100 g, respectively. These values represented an increase by 168.8%, 55.2%, and 49.7% when compared to sun drying, indicating that microwave pretreatment followed by rotary microwave vacuum drying could significantly enhance the retention of active components. As the temperature increased, the levels of chlorogenic acid, isochlorogenic acid A, loganin, and luteoloside initially increased and then decreased. This trend may be attributed to the high reactivity of these compounds, which are prone to oxidation, hydrolysis, and polymerization reactions as the temperature rises [47,48]. Additionally, at lower temperatures, enzymatic activity was higher, leading to prolonged drying times that are not conducive to the protection of these active components. The influence of different drying conditions on the three flavonoids, rutin, luteoloside, and quercetin, was relatively minor, and their individual trends and degrees of change varied. This variability may stem from the different numbers and arrangements of hydroxyl groups within each flavonoid compound, leading to differences in their degradation and loss during the drying process [49]. These findings are similar to those reported by Canale et al. [50].

### 3.4. Parameter Optimization Based on Entropy Weight TOPSIS Method

In the entropy weight method, evaluation indicators are divided into cost indicators and efficiency indicators, where smaller values indicate better cost performance and larger values indicate better efficiency performance [51]. The weight of each indicator is determined by its degree of dispersion. The higher the degree of dispersion, the greater the differentiation of the indicators, and the more information can be reflected, resulting in a higher assigned weight. The drying time was selected as the cost index, and its weight, obtained through the entropy weight method, was 0.0669. The efficiency indices, including chlorogenic acid, loganin, rutin, luteoloside, isochlorogenic acid A, isochlorogenic acid C, quercetin, total phenols, total flavonoids, polysaccharides, and antioxidant capacity, were selected as the efficiency indices. Their weights, obtained through the entropy weight method, were 0.0686, 0.0910, 0.0676, 0.0705, 0.0679, 0.0673, 0.1180, 0.0979, 0.1153, 0.1275, and 0.1083, respectively. Quercetin, total flavonoids, polysaccharides, and antioxidant capacity had relatively high weights, indicating that they were significantly influenced by different drying conditions during microwave pretreatment and rotary microwave vacuum drying. By using the TOPSIS comprehensive evaluation method, the multi-objective optimization problem was transformed into a single-objective optimization problem with the comprehensive evaluation value as the single index. This approach yielded the closeness coefficients D+ and D− for the evaluation indices and the optimal solutions under different drying parameters for *Honeysuckle*, as shown in Table 7. This indicates that at 50 °C, 35 Hz, and −0.065 MPa, the dried *Honeysuckle* achieved the highest comprehensive score, suggesting that under these drying conditions, the quality of the *Honeysuckle* was optimal.

## 4. Conclusions

To investigate the optimal pretreatment method for *Honeysuckle* among microwave, steam, and hot-blanching pretreatments, and using the sample treated with the best pretreatment method as the research subject, we studied the effects of different drying conditions during the process of rotary microwave vacuum drying on the drying rate and quality of *Honeysuckle*. The results are as follows: (1) The analysis of the effects of microwave, steam, and hot-blanching pretreatments on the overall quality of *Honeysuckle* indicated that all three pretreatments could inhibit enzymatic oxidation and effectively preserve the total phenolic and flavonoid compounds. However, microwave pretreatment showed significantly higher retention rates for total phenols, polysaccharides, antioxidant capacity, chlorogenic acid, and isochlorogenic acid A compared to the other pretreatment methods. Additionally, it resulted in a vibrant green color (L = 79.7 ± 0.16, a = −7.19 ± 0.07) and shorter drying times, making it the optimal pretreatment method. (2) The combined process of microwave pretreatment followed by rotary microwave vacuum drying reduced drying time and enhanced drying efficiency significantly. Both drying temperature and vacuum level had a substantial impact on the drying characteristics. Higher temperatures and lower vacuum levels led to faster drying rates. However, excessively high vacuum levels could prolong the machine’s heating-up time, resulting in reduced drying rates. (3) Compared to sun drying, microwave pretreatment followed by rotary microwave vacuum drying better preserved the quality of *Honeysuckle* flowers. Under different processing conditions, the maximum levels of total phenols, total flavonoids, polysaccharides, and antioxidant capacity could reach 54.73 mg/g, 71.14 mg/g, 268.67 mg/g, and 72.65%, respectively. Additionally, the overall color difference was minimal, indicating low structural damage to the dried product, and it exhibited a high rehydration ratio of 5.45. (4) Based on the entropy weight TOPSIS method using drying time and quality of *Honeysuckle* as criteria, the optimal drying parameters were determined to be a drying temperature of 50 °C, a vacuum level of −0.065 MPa, and a rotation speed of 35 Hz, which resulted in the highest comprehensive score for the dried product.

## Figures and Tables

**Table 1:** Browning situation under different pretreatment conditions.

Drying Condition	30 s	45 s	60 s	75 s
Microwave Pretreatment	Browning severe	Browning reduced	Browning reduced	Almost no browning
Steam Pretreatment	Browning severe	Browning reduced	Almost no browning	Almost no browning
Hot Water Blanching Pretreatment	Browning severe	Browning reduced	Almost no browning	Almost no browning

**Table 2:** Experimental factors for microwave vacuum drying.

Drying Temperatures (°C)	Vacuum (MPa)	Rotation Speed (Hz)
40	−0.060	20
50	−0.065	35
60	−0.070	50

Note: The rotating speed of rotor-type microwave vacuum drying rotor is expressed by frequency: rotation speed = 2π × frequency.

**Table 3:** Effect of different pretreatment methods on the active ingredients.

Drying Conditions	Organic Acids (mg/g)			Flavonoids (mg/100 g)			Diterpenes (mg/g)
	Chlorogenic Acid	Isochlorogenic Acid A	Isochlorogenic Acid C	Luteoloside	Rutin	Quercetin	Loganin
Microwave Pretreatment	44.73 ± 0.34 ^a^	11.24 ± 0.21 ^a^	2.22 ± 0.08 ^b^	56.34 ± 0.42 ^a^	19.23 ± 0.29 ^ab^	17.74 ± 0.11 ^b^	2.84 ± 0.05 ^a^
Steam Pretreatment	38.56 ± 0.08 ^b^	9.55 ± 0.16 ^b^	2.47 ± 0.33 ^b^	54.23 ± 0.88 ^a^	20.08 ± 0.44 ^a^	16.75 ± 0.20 ^b^	2.65 ± 0.07 ^a^
Hot Water Pretreatment	34.78 ± 0.07 ^c^	8.09 ± 0.11 ^c^	3.46 ± 0.10 ^a^	37.1 ± 0.27 ^b^	18.26 ± 0.46 ^b^	16.35 ± 1.39 ^b^	2.23 ± 0.13 ^b^
No Pretreatment	18.95 ± 1.22 ^d^	3.38 ± 0.24 ^d^	0.63 ± 0.04 ^c^	36.05 ± 3.25 ^b^	16.47 ± 0.83 ^c^	20.24 ± 0.78 ^a^	1.44 ± 0.20 ^c^

Note: A different lowercase letter after each column indicates a significant difference (*p* < 0.05).

**Table 4:** Effect of different pretreatment methods on the color.

	*L*	*a*	*b*	∆*E*
Microwave Pretreatment	79.7 ± 0.16 ^a^	−7.19 ± 0.07 ^c^	25.86 ± 0.17 ^c^	8.46 ± 0.15 ^b^
Steam Pretreatment	78.79 ± 0.10 ^b^	−6.14 ± 0.11 ^b^	26.87 ± 0.33 ^b^	8.14 ± 0.17 ^b^
Hot Water Pretreatment	76.94 ± 1.24 ^cd^	−6.73 ± 0.33 ^b^	25.88 ± 0.45 ^c^	7.99 ± 0.75 ^a^
No Pretreatment	77.49 ± 0.14 ^bc^	−5.09 ± 0.09 ^a^	25.64 ± 0.30 ^c^	9.32 ± 0.23 ^b^
Fresh	75.89 ± 0.39 ^d^	−11.97 ± 0.26 ^d^	31.71 ± 0.15 ^a^	-

Note: A different lowercase letter after each column indicates a significant difference (*p* < 0.05).

**Table 5:** Color and rehydration ratio parameters under different drying conditions.

Drying Conditions	*L*	*a*	*b*	∆*E*	Rehydration Ratio (*R*_f_)
Fresh	75.89 ± 0.39 ^f^	−11.97 ± 0.26 ^f^	31.71 ± 0.15 ^a^	-	-
Sun	83.41 ± 0.10 ^a^	−5.16 ± 0.07 ^b^	22.29 ± 0.19 ^e^	13.84 ± 0.21 ^a^	4.65 ± 0.06 ^e^
40 °C/35 Hz/−0.060 MPa	78.79 ± 0.46 ^de^	−6.52 ± 0.29 ^cd^	25.84 ± 0.18 ^d^	8.53 ± 0.28 ^cd^	5.27 ± 0.06 ^b^
50 °C/35 Hz/−0.060 MPa	79.70 ± 0.16 ^b^	−7.19 ± 0.07 ^e^	25.86 ± 0.17 ^d^	8.46 ± 0.15 ^cd^	5.29 ± 0.02 ^b^
60 °C/35 Hz/−0.060 MPa	79.34 ± 0.32 ^cd^	−6.93 ± 0.25 ^e^	26.68 ± 0.45 ^bc^	7.92 ± 0.58 ^e^	5.45 ± 0.04 ^a^
50 °C/20 Hz/−0.060 MPa	79.19 ± 0.45 ^cde^	−7.11 ± 0.24 ^e^	26.48 ± 0.21 ^bc^	7.87 ± 0.47 ^e^	5.25 ± 0.03 ^bc^
50 °C/50 Hz/−0.060 MPa	79.69 ± 0.13 ^b^	−7.14 ± 0.03 ^e^	26.33 ± 0.31 ^cd^	8.38 ± 0.15 ^de^	5.11 ± 0.03 ^d^
50 °C/35 Hz/−0.065 MPa	79.68 ± 0.36 ^bc^	−6.43 ± 0.04 ^c^	25.85 ± 0.01 ^d^	8.92 ± 0.12 ^c^	5.26 ± 0.02 ^bc^
50 °C/35 Hz/−0.070 MPa	78.88 ± 0.33 ^de^	−6.86 ± 0.10 ^de^	25.95 ± 0.13 ^d^	8.27 ± 0.11 ^de^	5.19 ± 0.04 ^c^

Note: A different lowercase letter after each column indicates a significant difference (*p* < 0.05).

**Table 6:** Content of active ingredients under different drying conditions.

Drying Conditions	Organic Acids (mg/g)			Flavonoids (mg/100 g)			Diterpenes (mg/g)
	Chlorogenic Acid	Isochlorogenic Acid A	Isochlorogenic Acid C	Luteoloside	Rutin	Quercetin	Loganin
40 °C/35 Hz/−0.060 MPa	38.62 ± 0.32 ^e^	10.03 ± 0.47 ^c^	2.29 ± 0.13 ^ab^	54.87 ± 0.12 ^b^	18.96 ± 0.49 ^b^	16.96 ± 0.36 ^f^	2.25 ± 0.04 ^d^
50 °C/35 Hz/−0.060 MPa	44.73 ± 0.34 ^a^	11.24 ± 0.21 ^a^	2.22 ± 0.08 ^ab^	56.34 ± 0.42 ^a^	19.23 ± 0.29 ^ab^	17.74 ± 0.11 ^de^	2.84 ± 0.05 ^a^
60 °C/35 Hz/−0.060 MPa	40.23 ± 0.71 ^d^	10.63 ± 0.19 ^b^	2.14 ± 0.14 ^b^	54.11 ± 0.28 ^bc^	19.44 ± 0.09 ^ab^	18.41 ± 0.46 ^b^	2.59 ± 0.03 ^bc^
50 °C/20 Hz/−0.060 MPa	41.38 ± 0.58 ^c^	10.68 ± 0.21 ^b^	2.18 ± 0.07 ^b^	51.29 ± 1.14 ^e^	19.48 ± 0.2 ^ab^	18.06 ± 0.22 ^bcd^	2.66 ± 0.15 ^b^
50 °C/50 Hz/−0.060 MPa	42.64 ± 0.47 ^b^	11.41 ± 0.023 ^a^	2.36 ± 0.16 ^a^	53.23 ± 1.45 ^cd^	19.65 ± 0.05 ^a^	18.07 ± 0.08 ^bc^	2.35 ± 0.03 ^d^
50 °C/35 Hz/−0.065 MPa	39.5 ± 0.84 ^de^	10.65 ± 0.16 ^b^	2.17 ± 0.05 ^ab^	50.76 ± 0.56 ^e^	19.63 ± 0.08 ^a^	17.54 ± 0.04 ^e^	2.26 ± 0.04 ^d^
50 °C/35 Hz/−0.070 MPa	43.79 ± 0.84 ^a^	11.44 ± 0.06 ^a^	2.24 ± 0.11 ^ab^	51.9 ± 0.33 ^de^	19.76 ± 0.08 ^a^	17.83 ± 0.06 ^cde^	2.49 ± 0.05 ^c^
Sun	16.64 ± 0.08 ^g^	3.40 ± 0.03 ^d^	0.64 ± 0.09 ^c^	37.64 ± 0.46 ^g^	14.72 ± 0.06 ^c^	19.39 ± 0.38 ^a^	1.83 ± 0.06 ^e^

Note: A different lowercase letter after each column indicates a significant difference (*p* < 0.05).

**Table 7:** Entropy weight TOPSIS method comprehensive score.

Drying Parameters	Positive Ideal Solution Distance (D+)	Negative Ideal Solution Distance (D−)	Relative Closeness	Sorting Results
40 °C/35 Hz/−0.060 MPa	0.200	0.180	0.473	7
50 °C/35 Hz/−0.060 MPa	0.087	0.279	0.763	1
60 °C/35 Hz/−0.060 MPa	0.101	0.240	0.703	2
50 °C/20 Hz/−0.060 MPa	0.139	0.226	0.619	4
50 °C/50 Hz/−0.060 MPa	0.142	0.212	0.600	5
50 °C/35 Hz/−0.065 MPa	0.182	0.178	0.495	6
50 °C/35 Hz/−0.070 MPa	0.123	0.238	0.659	3
Sun	0.285	0.122	0.301	8

## Data Availability

The original contributions presented in the study are included in the article, further inquiries can be directed to the corresponding author.
